# Phosphorylation of RIAM Activates Its Adaptor Function in Mediating Integrin Signaling

**DOI:** 10.33696/signaling.2.041

**Published:** 2021

**Authors:** Baihao Su, Jinhua Wu

**Affiliations:** Molecular Therapeutics Program, Fox Chase Cancer Center, Philadelphia, PA 19111, USA

**Keywords:** RIAM, Integrin, Phosphorylation, RAP1, FAK, SRC, Lamellipodin

## Abstract

Integrins are cellular receptors that regulate cell adhesion and many other cellular functions. Integrins can be activated via an “inside-out pathway” that is promoted by RAP1 GTPase. RAP1-GTP-Interacting Adaptor Molecular (RIAM) mediates integrin activation by linking RAP1 GTPase to talin, an integrin activator. RIAM’s function in integrin signaling is tightly regulated. In this commentary, we review recent studies of the molecular mechanisms underlying RIAM autoinhibition via both intramolecular interaction and oligomer assembly, and the phosphorylation-dependent activation of RIAM.

Extracellular matrix (ECM) surrounding cells in solid tissue provides mechanical support and communicates with cells through cell adhesion molecules (CAMs) at the cell surface [1,2]. Integrin is a major group of CAMs represented by a family of αβ heterodimeric single transmembrane receptors [3]. At least 18 integrin α subunits and eight integrin β subunits have been identified. Particular pairs of α and β subunits generate 24 distinct integrin species in vertebrates [3]. While some integrin species, such as β1 and α_v_ integrins, are ubiquitously expressed, others are expressed in a tissue-specific manner. In particular, β2 integrins are expressed exclusively on lymphocytes, whereas α_IIb_β_3_ is strictly expressed on platelets [3]. Hence, abnormal expression and mis-regulated activity of integrins may lead to thrombotic disorders, cardiovascular diseases, T cell proliferation defects, and many autoimmune diseases [4,5]. Integrins are emerging as appealing targets for therapeutic intervention, primarily by antagonizing extracellular ligand binding [4]. Indeed, two integrin inhibitors, Natalizumab and Vedolizumab, have been approved for treating inflammatory bowel diseases [6,7].

Conformational change of the ectodomains from a low-affinity binding state to a high-affinity binding state is a hallmark of integrin activation [8-10]. Two 4.1-ezrin-radixin-moesin (FERM) domain-containing proteins, talin and kindlin, govern this event in response to the activation of a small GTPase, Ras-associated protein 1 (RAP1) [11-13]. These two activators interact with the cytoplasmic tails of the integrin β subunit, leading to conformational change in the integrin ectodomains for ligands binding [14]. This signaling cascade from RAP1 to integrin is known as the “inside-out” integrin signaling pathway. In lymphocytes, the interaction of talin and integrin is promoted by a RAP1 effector known as Rap1-GTP-Interacting Adaptor Molecule (RIAM) [2,15]. RIAM is recruited to the plasma membrane (PM) by GTP-bound, active RAP1, and subsequently recruits talin to the PM where talin activates integrin. Upon activation, integrin engages with the ECM ligands and modulates cytoskeleton remodeling through an “outside-in” signaling pathway by activating downstream signaling molecules such as Focal adhesion kinase (FAK), integrin-linked kinase (ILK), and Src, leading to a multitude of cellular processes including cell migration, survival, and proliferation [16-19]. In this commentary, we review recent findings regarding the molecular mechanisms underlying RIAM autoinhibition and activation via phosphorylation in the “inside-out” signaling pathway.

## RIAM Mediates Integrin Activation

RIAM is abundantly expressed in leukocytes and is essential for the signaling of leukocyte-specific integrins. In particular, T cells highly express two types of β integrins that include VLA-4 (α_4_β_1_), α_5_β_1_ and α_6_β_1_ of β1 integrins and LFA-1 (α_L_β_2_) of β2 integrins [20]. RIAM has been shown to regulate the activation of VLA-4 and LFA-1 [21,22], the two integrins that play important roles in leukocyte trafficking during immune response [23,24]. RIAM has also been shown to regulate actin dynamics in T cells through Enabled/Vasodilator-stimulated phosphoprotein (Ena/VASP) family proteins and profilin [25]. Indeed, conventional T cells derived from RIAM knock-out mice exhibit impaired adhesion and defect of lymphocytes trafficking [21]. It is worth noting that RIAM’s function in regulatory T cells, when knocked out, can be compensated by a RIAM paralogue in mammalian known as lamellipodin (Lpd) [26], suggesting a rescue mechanism by regulating the expression level of paralogues. Subcellular localization of RIAM is dependent on the activation states of cells and cell types. RIAM is concentrated at the PM and cytoskeleton upon T cell activation [2,27]. RIAM is also recruited to the tips of lamellipodia when overexpressed in B16-F1, Swiss 3T3, and HEK293 cells [25,28], and to both lamellipodial tips and focal adhesions in stimulated NIH3T3 cells [28].

**Figure 1:**
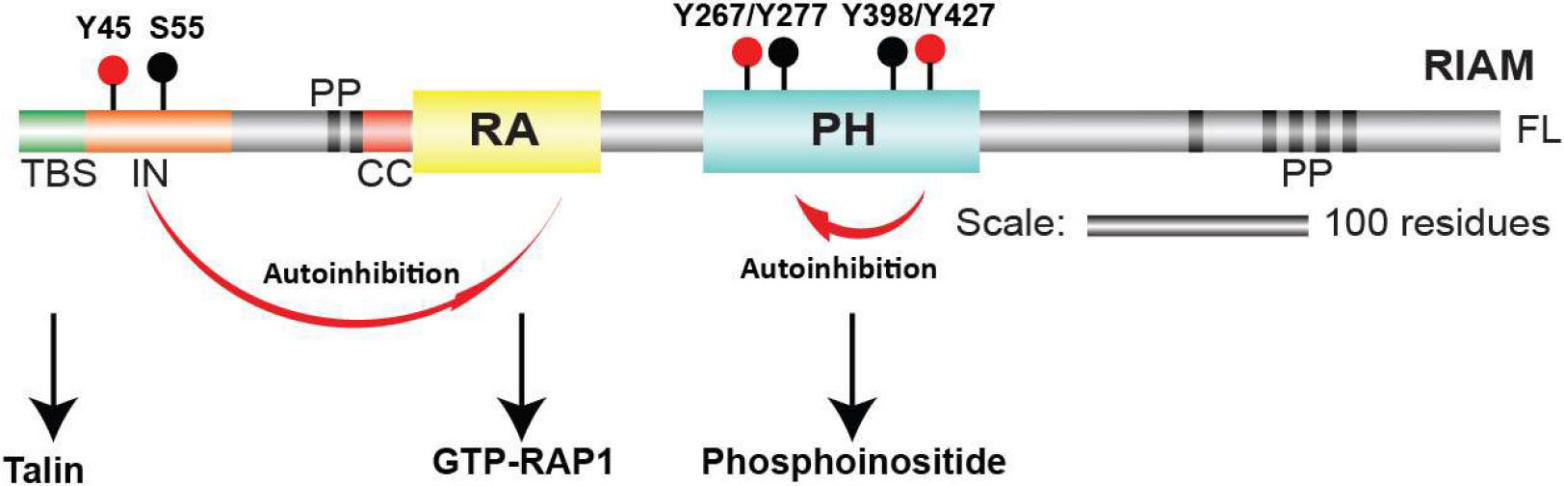
Schematic representation of RIAM. The talin-binding segment (TBS) is colored in green; inhibitory (IN) segment is in orange; coiled-coil (CC) segment is in red; poly-proline (PP) segment is in black; the Ras-associating domain (RA) is in yellow; and the pleckstrin homology domain (PH) is in cyan. The interaction of RA domain with GTP-bound RAP1 is inhibited by the IN segment, and the interaction of PH domain with phosphoinositide on the plasma membrane is inhibited by the PH domain from another RIAM molecule. Tyrosine phosphorylation sites that activate RIAM are indicated by red dots; those did not impact RIAM activity, and Ser55, are indicated by black dots.

RIAM is a RAP1 effector and mediates the “inside-out” integrin activation by activating talin and recruiting it to the PM [29,30]. The central structural module of RIAM accommodates a Ras-association (RA) domain that binds to RAP1 and a Pleckstrin-homology (PH) domain that engages with the PM. The two domains tightly contact each other through a hydrophobic interface and form a stable “RA-PH” structure module that are also seen in the Grb family adapter proteins [31]. At the amino side to the RA-PH, RIAM possesses a negative charged talin-binding segment (TBS), followed by an autoinhibitory segment (IN), two poly-proline (PP) motifs, and a coiled-coil (CC) region adjacent to the RA domain (Figure 1). The carboxyl-terminal side of RA-PH is largely disordered with multiple poly-proline (PP) motifs for EVH1 domain and profilin binding [25]. Upon RAP1 activation, the RA-PH module drives RIAM to the PM by associating with GTP-bound RAP1 via the RA domain, and with phosphoinositol 4,5-bisphosphate (PI(4,5)P_2_) in the PM via the PH domain [2]. Subsequently, RIAM recruits talin to the PM through the TBS segment by interacting with talin in the rod region [32]. RIAM also activates talin by an interaction of TBS with talin F3 subdomain in the head region. This interaction occludes the association of the F3 subdomain with the autoinhibitory domain in talin rod region, allowing the talin head F3 subdomain to interact with integrin β subunit [33].

## Phosphorylation in Integrin Signaling

The structural properties and the resulting activity states of these signaling proteins are often controlled by various types of post-translational modifications (PTMs). Among all PTMs, phosphorylation is the most commonly observed event in the integrin signaling pathway. The earliest studies of phosphorylation events in the integrin signaling pathway can be traced back to the 1980s [34-36]. It has been shown that phosphorylation of talin and kindlin regulates their functions in mediating integrin activation, and may alter their binding preference to certain integrin species [37-39]. Among other kinases, FAK and Src family kinases have been well documented for their roles in integrin signaling pathway.

FAK was known for its function of regulating signaling downstream of integrin activation. Integrin-mediated cell adhesion to ECM relieves FAK from autoinhibition, allowing phosphorylation of its activation loop [40,41]. Activated FAK interacts with intracellular signaling molecules such as Src, PI3-K, Grb7, thereby regulating various cellular functions including cell growth, cell migration, and intracellular trafficking. Src kinases have been shown to phosphorylate integrin and related adaptor proteins. The Spleen tyrosine kinase (Syk), a Src-family kinase, phosphorylates β3 integrin at the kindlin-binding site [42]. A lymphocyte-specific Src kinase, LCK, phosphorylates Lymphocyte function-associated antigen 1 (LFA-1, α_L_β_2_ integrin) and triggers downstream signaling that activates Phosphoinositide-3 kinase (PI3-K) and Syk [43]. In addition, Src also phosphorylates kindlin-2, vinculin, and tensin that play essential roles in promoting integrin-mediated focal adhesion and cell spreading [44-48]. Recently, both FAK and Src kinases have been reported to regulate integrin signaling by activating distinct functions of RIAM [49,50].

## RIAM Autoinhibition is Relieved by FAK and Src Phosphorylation

RIAM adapts an autoinhibitory conformation in which the IN segment binds to the RA domain. The IN:RA interface involves multiple side chain interactions that include Tyr45, Glu60, and Asp63 in the IN segment, and is further stabilized by the CC segment that resides on top of the IN segment [50]. Both Glu60 and Asp63 form salt bridges with Lys213 in the α1 helix of the RIAM RA domain. In the RAP1: RIAM complex structure, Lys213 of RIAM forms a signature interaction with Asp33 of RAP1, and a K213A mutation abolished the RAP1:RIAM interaction [51]. Thus, the IN segment masks the RAP1-binding site in the RA domain, resulting in autoinhibition of the RIAM RA domain. Releasing of this autoinhibition of RIAM for RAP1 binding requires phosphorylation at Tyr45. The bulky and negatively charged phosphoryl group of the pTyr45 disrupts the compact autoinhibitory interface, thus exposing the RA domain for RAP1 binding. Indeed, the phosphomimetic mutant Y45E or an interface mutation E60A/D63A enhance the *in vitro* RAP1:RIAM interaction and the PM co-localization of RAP1 and RIAM. Bioinformatic analyses suggest that Tyr45 is a substrate of FAK kinase, which was further confirmed by phosphorylation analyses using cells treated with FAK-specific inhibitors [50]. It is noteworthy that FAK is well known for its function in mediating “outside-in” integrin signaling at focal adhesions upon binding of integrins to the extracellular matrix. This new finding of FAK-dependent RIAM activation, therefore, reveals a crosstalk between “outside-in” and “inside-out” integrin signaling, by which integrin activation is amplified through a feedforward mechanism. In addition to Tyr45, endogenous phosphorylation of Ser55 in the IN segment has also been reported [52]. We have shown that RIAM bearing a phosphomimetic mutant of Ser55 also escapes from RA domain autoinhibition. Nevertheless, no notable change in the phosphorylation levels of Ser55 in anti-CD3 antibody stimulated Jurkat T cells was detected by proteomic analysis, suggesting that RIAM activation via Ser55 phosphorylation may be independent of T cell activation [50].

Besides the RA domain, the RAP1-dependent RIAM translocation to the PM also requires the PH domain to engage PI(4,5)P_2_ in the PM [2]. While the RA domain is autoinhibited via an intra-molecular interaction prior to RIAM activation, the PH domain is also autoinhibited via an inter-molecular interaction that blocks its PI(4,5) P_2_-binding site. This autoinhibitory interaction of the PH domain was first revealed by structural analyses and further validated by biochemical and functional characterization. Five crystal structures that contain the RA-PH module of RIAM have been reported [49]. These include a RA-PH alone structure (PDB: 6OLU), two structures of RA-PH with the CC segment (cc-RA-PH) in different crystal forms (PDB: 3TCA and 6O6H), a RA-PH:RAP1 complex structure (PDB: 4KVG), and an intramolecularly autoinhibited structure (IN-CC-RA-PH, PDB: 6E31) [2,49,50,53]. These structures were analyzed for conserved crystal packing interfaces to identify putative dimer assembly. While two of the five structures (PDBs: 3TCA and 4KVG) possess a common pseudo-symmetric intermolecular interface, all five structures share a conserved asymmetrical interface. It has long been suspected that RIAM adopts a dimer (or higher oligomer) state as found in its signaling partner talin and its mammalian paralogue Lpd [54,55]. These two conserved crystal contacts, both mediated by the PH domain, may represent dimer configurations of RIAM. We assessed RIAM oligomerization in a co-immunoprecipitation (co-IP) assay using full-length RIAM with two different tags (HA and GFP). Although the pseudo-symmetric interface is significantly larger than the asymmetrical interface, no notable effect in the oligomerization state of RIAM was observed in the pseudo-symmetric interface mutations (H389A/Y398A), suggesting that this interface is not physiologically relevant but is only a result of crystal packing. In contrast, RIAM A435Y mutation that disturbs the asymmetrical interface exhibited reduced dimerization in the co-IP assay, suggesting that the asymmetrical interface formed by the PH domain helical extension from one molecule and two β strains of another PH domain mediates RIAM dimerization [49]. Interestingly, the helical extension is only seen in the PH domain of RA-PH-containing proteins such as Grb7/10/14, and has also been shown to mediate the dimerization of Grb10 [56]. RIAM dimerization was further validated by size exclusion chromatography (SEC) analyses. Nevertheless, our unpublished work indicated no dimer formation of recombinant RA-PH alone in solution, suggesting that other regions of RIAM are also required to stabilize the dimer configuration of RA-PH.

Masking of the PI(4,5)P_2_-binding site in the dimer configuration of RIAM RA-PH results in the suppression of the PM translocation of RIAM. The two β strains of the neighboring PH domain that interact with the helical extension accommodate a positively charged patch composed of Lys327, Lys328, Lys331, Arg332, and Arg333 that engages with PI(4,5)P_2_ in the PM in response to RAP1 activation [2]. This PI(4,5)P_2_-binding site needs to be unmasked for RIAM translocation to the PM. Indeed, RIAM-A435Y, the helical extension mutation that disrupts dimer assembly and unmasks the PI(4,5)P_2_-binding site, exhibited enhanced PM translocation [49]. Consequently, RIAM-A435Y up-regulates integrin activity in CHO-A5 cells that stably express α_IIb_β_3_ integrin. Similar to the RA domain autoinhibition, this dimerization-induced inhibition of PH: PI(4,5)P_2_ interaction is also regulated by phosphorylation. RIAM dimerization is significantly reduced upon phosphorylation as indicated by the co-IP assay. Moreover, RIAM is highly phosphorylated during T cell activation, and the phosphorylation of RIAM is coupled with its PM translocation, consistent with the model that phosphorylation of RIAM disrupts RIAM dimerization, in turn promotes its association with the PM. Further analyses revealed that RA-PH module is the substrate of Src family kinases. An LCK inhibitor, but not FAK inhibitors, suppressed the phosphorylation of the RA-PH module of RIAM upon T cell activation [49]. The LCK inhibitor (RK-24466) also suppressed the PM translocation of RIAM in Jurkat T cells and LFA-1-mediated T cell adhesion [12]. Mass spectrometry analyses revealed four tyrosines that are highly phosphorylated by LCK and Fyn, two Src family kinases abundant in lymphocytes. Among the four tyrosines, Tyr267 and Tyr427 reside near the dimer interface, and Tyr398 is located in the non-physiological, crystal packing interface, whereas Tyr277 is in a disordered loop. Therefore, phosphomimetic mutations of Tyr267 and Tyr427 (Y267E, Y427E, or Y427E/Y427E) were expected to diminish the dimer assembly via the PH domain. Indeed, these mutations promoted RIAM PM localization in both Jurkat T cells and HEK293T cells. In contrast, the nonphosphorylable mutations, Y267F or Y427F, severely suppressed RAP1-mediated RIAM PM localization as the RA-PH module is locked in the inhibitory dimer assembly. Together, these results indicated that the phosphorylation of Tyr267 and Tyr427 in RIAM RA-PH by Src family kinases unmasks the PI(4,5)P_2_-binding site in RIAM PH domain, thus promoting the PM translocation of RIAM and subsequent integrin activation [49].

To our knowledge, this is the first example of such a regulatory mechanism of a PH domain, in which the exposure of PI(4,5)P_2_-binding site in the PH domain is regulated by homo-oligomerization and phosphorylation. Nevertheless, bioinformatic analyses of crystal structures of PH domains using ProtCID revealed that Lpd may also possess a similar oligomer assembly that masks the PI(4,5)P_2_ binding site [49]. The RA-PH module in Lpd can be phosphorylated by Abl and Src kinases. *In vitro* kinase assays using synthetic peptides derived from Lpd suggested six tyrosines in the PH domain that were phosphorylated by both Abl and Src [57,58]. Structural alignment of the PH domains of Lpd and RIAM reveals that four of the six sites correspond to the phosphorylation sites in the RIAM PH domain including Lpd-Tyr357 (RIAM-Tyr207), Lpd-Tyr366 (RIAM-Tyr277), Lpd-Tyr481 (RIAM-Tyr398), and Lpd-Tyr510 (RIAM-Tyr427) [2,54], supporting our speculation that the PI(4,5)P_2_-binding site in Lpd may be regulated by a similar, phosphorylation-dependent mechanism. Moreover, Lpd’s function in cell motility is regulated by phosphorylation mediated by Abl and Src. Lpd interacts with both the EVH1 domain of Ena/VASP and Abi of the SCAR/WAVE complex via the C-terminal polyproline motifs. Although the binding specificity to EVH1 or Abi of these polyproline motifs remains unclear, the interaction of Lpd with Ena/VASP or with SCAR/WAVE complex is controlled by Lpd phosphorylation. Nonetheless, phosphorylation of Lpd by Src only promotes its association with the SCAR/WAVE complex, while phosphorylation of Lpd by Abl promotes the interaction of Lpd and Ena/VASP and its association with the SCAR/WAVE complex [57,58]. The *in vitro* kinase assays suggest similar phosphorylation patterns of Lpd in the C-terminal region by Abl and Src kinases [57,58]. In cells, however, phosphorylation levels of these sites in the context of the full-length Lpd by Abl and Src may be significantly different due to local structural environment of the substrate motifs and cellular or subcellular abundance of the kinases. Thus, analyses of cell-based phosphorylation of properly folded Lpd are needed to elucidate the molecular mechanisms underlying this effector binding selectivity.

## Other Forms of Regulation in Integrin Signaling

**Figure 2:**
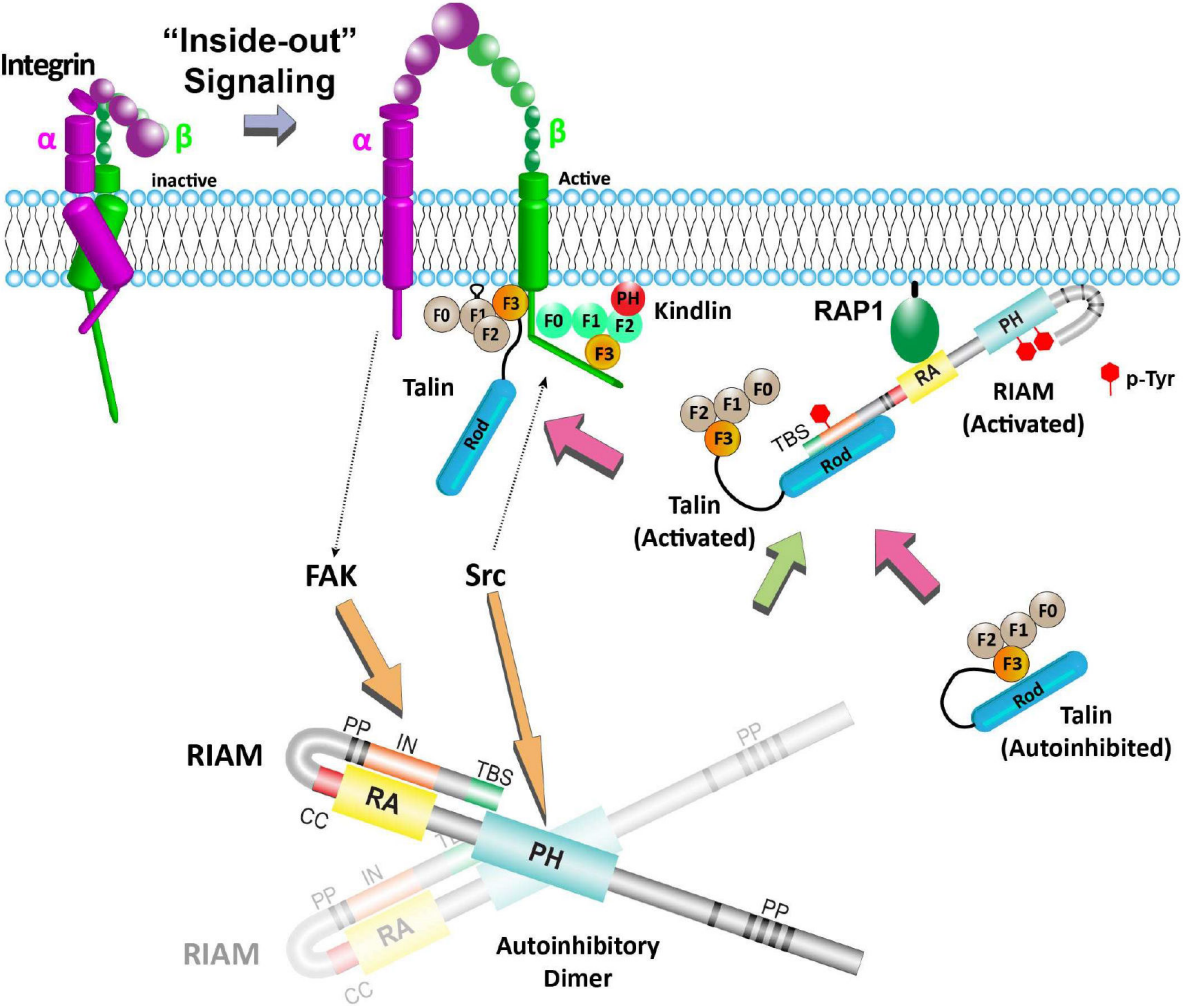
Phosphorylation of RIAM activates its function in mediating integrin activation. The RA-PH module of RIAM is autoinhibited in a dimer assembly. The color scheme of RIAM is the same as Figure 1. This autoinhibitory dimer configuration is released upon phosphorylation by FAK and Src kinases. Phosphorylated tyrosines in activated RIAM are indicated by red hexagons. The two kinases activate RIAM and promotes the PM translocation of RIAM by interacting with RAP1 and the PM. RIAM in turn recruits and activates talin. Binding of talin and kindlin to the β tail of integrin activates integrin.

Together, these recent studies demonstrate that both the RA and PH domains of RIAM are autoinhibited for its adaptor function in mediating integrin activity, and phosphorylation of RIAM mediated by FAK and Src kinases regulates RIAM activity (Figure 2). Other adaptors, such as kindlin and talin, have also been shown to promote integrin signaling upon phosphorylation [37,39]. Moreover, other types of PTMs were also found in integrin and related adaptors to regulate their cellular functions. Lysine acetylation in β1 integrin regulates fibronectin matrix assembly [59]. Ubiquitination of the α5 integrin regulates the subcellular trafficking of α5β1 integrin [60]. Proteolytic cleavage of talin and kindlin by calpain also regulates integrin-mediated cell adhesion [61,62]. In addition, vinculin has been shown to be acylated by both myristic acid and the palmitic acid with unknown functions [63,64]. These PTMs may regulate the functions of the adaptors by altering their structural configurations, binding properties, and homo-/hetero-oligomer assemblies.

In addition to PTMs, the function of the adaptors can also be modified prior to translation by RNA splicing, which often results in the loss of distinct functional modules in various isoforms. For instance, RIAM has a splicing isoform that lacks the PH domain and the C-terminal polyproline motifs [25], and Lpd possesses at least nine splicing isoforms, many lacking the entire C-terminal polyproline region [65]. One can speculate that the function of these shorter isoforms in regulating cytoskeletal rearrangement may be largely compromised. It is therefore imperative to devote more efforts on the understanding of the regulatory mechanisms driven by these PTMs and various isoforms, particularly in the signaling pathways mediated by integrins or small GTPases.
